# *e*cancer—towards effective global cancer control through innovation, collaboration and dissemination

**DOI:** 10.3332/ecancer.2019.ed90

**Published:** 2019-04-01

**Authors:** Eduardo Cazap

**Affiliations:** Editor-in-Chief, ecancer, 13 King Square Avenue, Bristol, BS2 8HU, UK; President, Latin American and Caribbean Society of Medical Oncology (SLACOM), Buenos Aires, Argentina

I am deeply privileged and proud on my appointment as *e*cancer’s Editor–in-Chief following two inspirational cancer leaders who founded *e*cancer twelve years ago: Professor Umberto Veronesi, a global leader who opened up new cancer treatment avenues in the world, and Professor Gordon McVie, the co-founding editor of *e*cancer.

The mission defined by the founders was to optimise patient care and outcomes by improving cancer communication and education, with no financial or geographical barriers. I strongly believe that education is a fundamental tool in obtaining better results for patients and new and better knowledge for doctors globally.

In my view, *e*cancer has two important characteristics. One is that *e*cancer is run by a charity, the *e*cancer Global Foundation, which supports a totally free platform. Additionally, the publication of scientific papers or related pieces of information is free for the authors. The number of publications, videos and other educational material daily downloaded from the platform around the world is gigantic.

I envision, together with the exceptional *e*cancer team, a new period of expansion in the coming years. Cancer control has become today one of the core principles in public policy. Non-communicable diseases (NCDs) are now included in the agendas of global cancer organisations and institutions.

Governments, civil society, non-governmental organisations, the private sector and patients are taking responsibility in this endeavour. Cancer is today a problem for the whole of society.

*e*cancer will reflect these important global movements in the material included in our platforms and media together with the existing information. We are planning to include new ideas and innovative projects of global leaders and organisations from around the world to share with our audience the current efforts ongoing in different countries and regions to improve the situation. We are also committed to include the voice of young leaders and scientists to know how they perceive the current picture and propose future actions.

A realistic way of doing all this work is being funded by charities and foundations. Funding for cancer control activities is scarce and the *e*cancer Global Foundation is supportive of these principles.

Cancer is a global challenge but the solutions must be provided at the local level. Regarding this particular point, we plan for the next two to four years new activities in India, Africa and also the expansion and promotion of our current activities in Latin America.

I am committed, together with the *e*cancer Global Foundation, *e*cancermedicalscience and the team in the UK, to contribute towards all of these efforts with all my capabilities.

I also expect the collaboration of good friends that I have around the world to help us in obtaining our goals.

Thank you very much.

